# Search for Wives

**Published:** 1861-08

**Authors:** H. Daniels


### SEARCH FOR WIVES.

BY REV. H. DANIELS.

Where do men usually discover the women who afterwards become their wives?—is a question we have occasionally heard discussed, and the custom has invariably become of value to young lady readers. Chance has much to do in the affair, but then there are important and governing circumstances. It is certain that few men make a selection from ball-rooms, or any other places of public gayety, and nearly as few are influenced by what may be called "showing off" in the streets, or by any allurements of dress. Our conviction is, that ninety-nine hundred parts of all the finery with which women decorate or load their persons go for nothing, as far as husband-catching is concerned. Where, and how, then, do men find their wives? In the quiet homes of their parents or guardians, at the fireside, where the domestic graces and feelings are alone demonstrated. These are the charms which most surely attract the high as well as the humble. Against these, all the finery and airs in the world sink into insignificance. We shall illustrate this by an anecdote.

A certain gentleman, whose health was rapidly declining, was advised by his physicians to try a change of climate as a means for recovering his health. His daughters feared that those who had only motives entirely mercenary, would not pay him that attention which he might expect from those who, from duty and affection united, would feel the greatest pleasure in ministering to his ease and comfort. They therefore resolved to accompany him. They proved that it was not a spirit of dissipation and gayety that led them to do this, for they were not to be seen in any of the gay and fashionable circles; they were never out of their father's company, and never stirred from home except to attend him, either to take the air, or drink the waters. In a word they lived a recluse life in the midst of a town then the resort of the most illustrious and fashionable personages of Europe. This exemplary attention to their father procured these three amiable sisters the admiration of all the English at S———, and was the cause of their elevation to that rank in life to which their merits gave them so just a title. They were all married to noblemen—one to the Earl of B———, another to the Duke of H———, and afterwards to the Marquis of E———, and a third to the Duke of N——— ; and it is justice to them to say, that they reflected honor on their rank, rather than derived any from it.

